# Bypassing Mechanisms of Mitochondria-Mediated Cancer Stem Cells Resistance to Chemo- and Radiotherapy

**DOI:** 10.1155/2016/1716341

**Published:** 2015-11-30

**Authors:** Alex Lyakhovich, Matilde E. Lleonart

**Affiliations:** ^1^International Clinical Research Center, St. Anne's University Hospital, Masaryk University, Kamenice 5/A7, 625 00 Brno, Czech Republic; ^2^Institute of Molecular Biology and Biophysics, Novosibirsk, Russia; ^3^Oncology and Pathology Group, Institut de Recerca Hospital Vall d'Hebron, Passeig Vall d'Hebron 119-129, 08035 Barcelona, Spain

## Abstract

Cancer stem cells (CSCs) are highly resistant to conventional chemo- and radiotherapeutic regimes. Therefore, the multiple drug resistance (MDR) of cancer is most likely due to the resistance of CSCs. Such resistance can be attributed to some bypassing pathways including detoxification mechanisms of reactive oxygen and nitrogen species (RO/NS) formation or enhanced autophagy. Unlike in normal cells, where RO/NS concentration is maintained at certain threshold required for signal transduction or immune response mechanisms, CSCs may develop alternative pathways to diminish RO/NS levels leading to cancer survival. In this minireview, we will focus on elaborated mechanisms developed by CSCs to attenuate high RO/NS levels. Gaining a better insight into the mechanisms of stem cell resistance to chemo- or radiotherapy may lead to new therapeutic targets thus serving for better anticancer strategies.

## 1. Introduction

One of the hypotheses explaining tumor progression suggests the existence of a group of cells with a stem phenotype which preserves tumors through a continuous production of progeny [1]. In recent years, the CSCs hypothesis has gained ground in several cancers [2]. The CSCs mediate tumor resistance to chemo- and radiation therapy and are also capable of invading and migrating to other tissues [3]. Similarly to cancer cells (CCs), the CSCs features include self-renewal capacity, the ability of proliferation, migration to and homing at distant sites, and resistance to toxic agents. Accordingly, CSCs identification and isolation include in vitro (sphere forming, Hoechst dye exclusion, aldehyde dehydrogenase ALDH enzymatic activity, surface markers, colony forming, lable retention, and migration) and in vivo (tumor propagation, xenotransplantation) assays. This theory has been recently supported by the findings that, among all malignant cells within a particular tumor, only CSCs have the exclusive potential to generate tumor cell population [4]. Given these shared attributes, cancer was proposed to originate from transforming mutation(s) in normal stem cells that deregulate their physiological programs [5]. In turn, intrinsic or acquired resistance of CSCs involves mechanisms such as genetic aberrations, quiescence, overexpression of drug transporters, DNA repair ability, and overexpression of antiapoptotic proteins [6]. Intrinsic resistance to chemotherapy is emerging as a significant cause of treatment failure and evolving research has identified several potential causes of resistance most of which end up in increased apoptosis [7]. Among the mechanisms of CSC-related therapy resistance may include ROS resistance, activation of ALDH, active developmental pathways (Wnt, Notch), enhanced DNA damage response, deregulated autophagy, altered metabolism, and microenvironmental conditions [8]. Surprisingly, most of the above-mentioned pathways in CSCs are mediated by redox misbalance and involvement of mitochondria-mediated antioxidant capacity [9].

The major exogenous source of reactive species in eukaryotic cells is mitochondria. In normal cells, RO/NS concentration is maintained at certain threshold required for signal transduction or immune response mechanisms, and CSCs, which exhibit an accelerated metabolism, demand high ROS concentrations to maintain their high proliferation rate [10]. The imbalance between ROS generation and detoxification, known as OS, is thought to be involved in cancer development and progression [11, 12].

**Figure 1:**
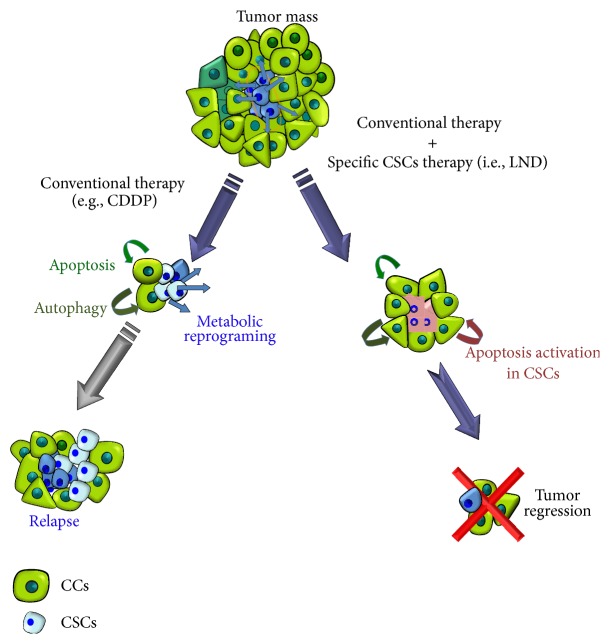
CSCs survival after chemo-/radiotherapy. The percentage of CSCs in a tumor varies depending on tumor type and tumor stage but generally comprises 0.5–5%. Most CCs in a tumor are killed after radiation or conventional chemotherapy (i.e., CDDP). The most important consequence of this is that although the tumor disappears in some cases (i.e., by image such us nuclear magnetic resonance), the percentage of CSCs has not diminished; quite the contrary it increased in proportion to the whole number of microscopically tumoral cells (reaching till 50% or more). CSCs left behind unaffected, due to their chemo- and radioresistance, eventually will experience metabolic reprograming to give rise to new CCs and CSCs, nesting the gap left by the tumor often with more aggressive phenotype. The cotreatment of conventional therapy with a more specific drug against CSCs (i.e., LND) in parallel will solve this problem.

Chemo-/radioresistance to cancer therapy is an unsolved problem in oncology [13]. Numerous studies have attempted to explain mechanisms of resistance over the last decades. CSCs may be innately resistant to many standard therapies due to a high antioxidant capacity and inability to perform apoptosis thus surviving cytotoxic or targeted therapies (Figure 1) [14]. Here we review the progress of CSCs studies made for the last years focusing on possible mechanisms of CSCs radio- and chemoresistance in connection to oxidative stress (OS) and summarizing some therapeutic approaches to overcome that issue.

## 2. Resistance of CSCs to Conventional Chemo- and Radiotherapeutic Regimes in Connection to Oxidative Stress (OS)

Although conventional chemotherapy kills most cells in a tumor, it is believed to leave CSCs behind causing chemo- and radioresistance (Table 2). As a consequence, CSCs persist in the body of cancer patients and in the middle-long term will migrate to the blood to nest in distal organs to metastasize. In the last five years, several protective CSC pathways have been proposed. The multifunctional efflux transporters from the superfamily of human ATP-binding cassette (ABC) are among them. They comprise seven subfamilies with 49 genes grouped into seven families (from A to G) with various functions, and at least 16 of these proteins are implicated in cancer drug resistance [15]. These ABC proteins have been known to also participate in multidrug resistance (MDR) of tumor cells [16]. Recent data demonstrate their role in protection of CSCs from chemotherapeutic agents [17]. Importantly, they are engaged in redox homeostasis and protection from OS in mammals [18]. Malfunction of the ABCD1 gene impairs oxidative phosphorylation (OXPHOS) triggering mitochondrial ROS production from electron transport chain complexes [19]. ABCC9 is required for the transition to oxidative metabolism [20]. Deficiency of a transregulator of mitochondrial ABC transporters PAAT decreases mitochondrial potential and sensitizes mitochondria to OS-induced DNA damages [21]. Drug resistance in colon CSCs is mediated by the ABC G member 2 (ABC-G2) and regulated by Ape1 redox protein [22]. Overall, one may conclude that redox dysregulation of one or several ABC members may significantly impact CSCs survival after chemotherapeutic treatment. Decreasing the activity of ABC transporters may therefore overcome drug resistance [23].

On the other hand, developmental pathways such us the Epithelial-Mesenchymal Transition (EMT) play crucial roles in tumor metastasis and recurrence. EMT process resembles very much the fate of CSCs and is involved in de novo and acquired drug resistance [24]. Altered production of RO/NS is involved in the regulation of CSC and EMT characteristics [25]. Moreover, microRNAs play also key roles in this aspect. For example, miR-125b suppressed EMT by targeting SMAD2 and SMAD4 [26]. Moreover, secreted frizzled-related protein 4 (sFRP4) chemosensitized CSC-enriched cells to the most commonly used antiglioblastoma drug, temozolomide (TMZ), by the reversal of EMT. Significantly, the chemosensitization effect of sFRP4 was correlated with the reduction in the expression of drug resistance markers ABCG2, ABCC2, and ABCC4 [27]. These findings could be exploited for designing better targeted strategies to improve chemoresponse and eventually eliminate CSCs.

## 3. Apoptosis and CSCs Resistance due to Increased Antioxidative Properties

An increasing number of conventional and novel generation chemotherapeutical drugs induce apoptosis through the induction of OS. If decreased RO/NS detoxification in CSCs is indeed a prime factor for chemo- or radioresistance prooxidant chemicals as, for example, malonohydrazides, targeting the redox state of pathogenic versus nonpathogenic cells may represent a challenging solution. The most developed drug of this class, STA-4783 (elesclomol), targets OS by Hsp70 induction and induces ROS within CCs [28]. Shepherdin is one of the first rationally designed mitochondrial drugs targeting Hsp90/TRAP1 functions through inhibiting ATPase activities. The tumor necrosis factor (TNF) receptor-associated protein 1 (TRAP1) is a mitochondrial homologue of Hsp90 [29]. Phosphorylation of TRAP1 by PTEN is responsible for the protection of ROS-mediated cell death [30]. Therefore, blocking the ATP pocket in the Hsp90 by shepherdin or geldanamycin causes inhibition of the TRAP1 chaperone function and may provide a novel strategy to design an anti-CSCs drugs [31]. SMIP004 (N-(4-butyl-2-methyl-phenyl) acetamide), a novel anticancer drug, induces mitochondrial ROS formation and disrupts the balance between redox and bioenergetics states [32].

Recent works by Kim et al. identified CD13(+) liver CSCs surviving in hypoxic lesions after chemotherapy, presumably through increased expression of CD13/aminopeptidase N, a ROS scavenger [45]. CD13 also enhances the generation and accumulation of mutations following DNA damage. Therefore, the CD13(+) dormant cancer stem cells must be eradicated fully to achieve complete remission of cancer [46]. The resistance of CD133 positive CSCs to chemotherapy can also be linked with higher expression of BCRP1 and MGMT, as well as the antiapoptosis protein and inhibitors of apoptosis protein families [47, 48].

Resistance of glioma to chemo- or radiotherapy is associated to inability of glioma CSCs to undergo apoptosis. Combined therapy aiming to inhibit AKT/mTOR signalling pathway and reactivate TP53 functionality allowed triggering cellular apoptosis [49]. Rottlerin (ROT) is widely used as a protein kinase C-delta (PKC-*δ*) inhibitor has been found to induce apoptosis via inhibition of PI3K/Akt/mTOR pathway and activation of caspase cascade in human pancreatic CSCs [33].

Nuclear factor erythroid 2-related factor 2 (Nrf2) is an essential component of cellular defense against a variety of endogenous and exogenous stresses [50]. NRF2 is an inducible transcription factor that activates a battery of genes encoding antioxidant proteins and phase II enzymes in response to oxidative stress and electrophilic xenobiotics [51]. NRF2-silencing in CSCs models, known as mammospheres, demonstrated increased cell death and lack of anticancer drug resistance [52]. Moreover, dedifferentiated cells upregulate MDR genes via Nrf2 signaling and suggest that targeting this pathway could sensitize drug-resistant cells to chemotherapy [53]. Interestingly, bardoxolone methyl (also known as CDDO-Me or RTA 402) is one of the derivatives of synthetic triterpenoids acting via Nrf2 and has been used for the treatment of leukemia and solid tumors [34].

## 4. Chemo- and Radioresistance of CSCs due to Impaired Autophagy: Novel Therapeutic Targets

Autophagy, also referred as “cell cannibalism,” is the degradation of cytoplasmic components, protein aggregates, and organelles through the formation of autophagosomes, which are degraded by fusion with lysosomes [54]. This process depends on a group of evolutionarily conserved autophagy-related (ATG) genes [55]. Although autophagy and apoptosis are apparently two different mechanisms, one promoting cell survival and the latter cell death, they are quite coordinated in the cells. For example, Beclin-1 (Bec1), the mammalian orthologue of yeast Atg6, is part of the class III phosphatidylinositol 3-kinase (PI3K) complex that induces autophagy. Beclin-1 interacts with the antiapoptotic protein Bcl-2 and its dissociation is essential for its autophagic activity [56].

Hypoxia-mediated autophagy has been previously suggested to promote the survival of CSCs of various origin. Hypoxia-inducible factor-1*α* (HIF-1*α*), one of the key players of cell survival response to hypoxia, was shown to convert non-stem pancreatic cancer cells into pancreatic cancer stem-like cells through autophagic mechanisms [57]. HIF1 induction and NF*κ*B activation are sufficient to induce the autophagic degradation of breast CSCs [58]. Inhibition of Wnt by resveratrol in breast CSc [35] and Notch by honokiol in melanoma SCs [36] suggests involvement of these autophagy-related players in regulation of CSCs signaling pathways.

Autophagy plays a critical role in adaptation to stress conditions in CCs and can enhance the radio- and chemoresistance of CSCs by limiting OS and protecting CSCs stemness properties [59]. Although mechanisms inducing autophagy are not fully understood, the connection of the CSCs resistance to the chemo- and radiotherapy is supported by a number of indirect evidences. Platin-derived drugs, which are used commonly in the conventional chemotherapeutical treatments, have a role in autophagy. For example, cisplatin (CDDP) preferentially induces autophagy in resistant esophageal CCs EC109/CDDP but not in EC109 cells (parental or sensitive to CDDP) [60]. Moreover, abolition of autophagy by pharmacological inhibitors or knockdown of ATG5/7 resensitized EC109/CDDP cells. In particular, the chemotherapeutic drug oxaliplatin induced autophagy, enriched the population of colorectal CSCs, and participated in maintaining the stemness of colorectal CSCs, thus making the cells more resistant to chemotherapy [61].

The Janus-activated kinase 2- (Jak2-) signal transducer and activator of transcription 3 signaling pathway may play a role in autophagy-dependent chemoresistance of CSCs derived from triple-negative breast tumors. In a recent study by Choi et al., chloroquine (CQ), an antimalarial reagent which blocks autophagy, was identified as a potential CSC inhibitor [37]. The CQ is known to evoke mitochondrial ROS and ROS scavengers may decrease CQ-induced mitochondrial autophagy [62]. All these facts support the note of ROS-dependent autophagic survival of CSCs. Recently explored inducible mouse model of mutated Kras revealed that a subpopulation of dormant tumor cells surviving oncogene ablation have features of CSCs and their tumor relapse is dependent on expression of genes governing OXPHOS, mitochondrial respiration, and autophagy [63].

Highly synergistic growth inhibition was observed in patient-derived lung CSCs exposed to a multitarget folate antagonist pemetrexed followed by a histone deacetylase inhibitor ITF2357, a known autophagy inducer [38]. A few studies using cultured cells found that melatonin promoted the generation of ROS at pharmacological concentrations [64]. Treatment with melatonin induced glioma CSCs death with ultrastructural features of autophagy [65].

Reduced glutathione (GSH) is considered to be one of the most important scavengers of reactive oxygen species (ROS), and its ratio with oxidised glutathione (GSSG) may be used as a marker of oxidative stress [39]. The side population (SP) cells from bladder cancer cell lines which resemble characteristics of CSCs had low ROS levels and high GSH/GSSG ratio and might contribute to radioresistance of CSCs [66]. The SP cells also showed substantial resistance to gemcitabine, mitomycin, and cisplatin compared with the non-SP counterparts and revealed a high autophagic flux associated with the ABCG2 expression. Importantly, pharmacological and siRNA mediated inhibition of autophagy potentiated the chemotherapeutic effects of gemcitabine, mitomycin, and CDDP in these CSCs. This may represent a potent target for the treatment of bladder carcinoma [67]. Screening studies by Jangamreddy et al. identified molecules that were preferentially toxic to CSCs, in particular, K+-ionophore salinomycin [40]. Salinomycin causes mitochondrial dysfunction, decreases ATP production, and induces autophagy [68]. Under hypoxia or/and low glucose level (the primary energy source for CCs) its toxicity towards CCs is amplified [69]. The mechanism includes activation of the AMP activated protein kinase (AMPK) that triggers autophagy making salinomycin to be anti-CSCs chemical [70]. The combination of AMPK agonist such as metformin and a glycolysis inhibitor 2-deoxyglucose (2DG) led to significant cell death associated with a sustained autophagy inhibiting tumor growth in mouse xenograft models [71]. Since AMPK activation was shown to mediate the metabolism reprogramming in drug-resistant CCs including promoting Warburg effects and mitochondrial biogenesis, both salinomycin and corresponding inhibitors of AMPK are now suggested to combat chemo- and radiotherapeutic resistance of CSCs [72].

Another type of selective autophagy, called mitophagy is served to the removal of dysfunctional mitochondria from the cells and is often controlled by moderate level of ROS [73, 74]. During mitophagy dysfunctional mitochondria are engulfed by a double-layered membrane (phagophore) that forms so-called autophagosome followed by degradation [75]. Among several drugs inducing mitophagy proton pump inhibitor ESOM damages mitochondria through NADPH oxidase and ROS accumulation [76]. The ESOM may work as a synthetic lethal reagent which increases cytotoxicity if used upon knockdown of Beclin-1 [77]. Another drug DCA (dichloroacetate) is a small molecule and a mitochondria-targeting agent. In CCs, the DCA induces mitophagy through accumulation of ROS and reduction of lactate excretion followed by the increase of NAD(+)/NADH ratio [78]. Importantly, paclitaxel-resistant cells contained sustained mitochondrial respiratory defect. DCA specifically acts on cells with mitochondrial respiratory defect to reverse paclitaxel resistance. DCA could not effectively activate oxidative respiration in drug-resistant cells but induced higher levels of citrate accumulation, which led to inhibition of glycolysis and inactivation of P-glycoprotein [79].

Overall, the above data provide multiple lines of evidence supporting the idea that impaired autophagy coupled with OS plays an essential role in the development of drug resistance, self-renewal, differentiation, and tumorigenic potentials of CSCs, implying the therapeutics potential of autophagy inhibitors to overcome that issue (Table 2).

## 5. OS, Mitochondria, and CSCs

In mammalian systems RO/NS presumably include so-called free (^•^OH, RO^•^, ROO^•^, NO^•^, hydroxyl, alkoxyl, peroxyl, and nitroxyl), superoxide (O_2_^•−^) radicals, and peroxides (H_2_O_2_, RO_2_H) and are mainly generated by OXPHOS in mitochondria, whereas, in pathological conditions, high level of RO/NS can be mitochondria dependent (ischaemia, loss of cytochrome c, low ATP demand and consequent low respiration rate, diabetes, DNA damage, and mutations), independent or indirect (cancers, tissue injuries, and inflammatory events) [80, 81]. Importantly, being the main source of RO/NS generation, mitochondria are also their primary and the most susceptible target. This may evoke a “secondary wave” of OS generated by damaged mitochondria followed by formation of extra RO/NS or by inhibition of detoxifying enzymes and generation more RO/NS flux thus forming a vicious cycle [82]. In fact, decreased mitochondrial priming in colon CSCs responsible for resistance to conventional chemotherapy has been recently determined [83]. The relevance of OXPHOS has also been shown in glioblastoma (GBM) sphere cultures (glioma spheres). Insulin-like growth factor 2 mRNA-binding protein 2 (IGF2BP2) expression provides a key mechanism to ensure OXPHOS maintenance by delivering respiratory chain subunit-encoding mRNAs to mitochondria and contributing to complex I and complex IV assembly [84]. Several antioxidant enzymes such as Mn, Cu, Zn-containing superoxide dismutases (SODs), glutathione peroxidase, glutathione reductase (GPx), glutathione S-transferase (GSTs), and catalase protect DNA from OS [85]. Unlike CSCs, CCs have higher bioenergetic metabolism, higher ROS level, and higher capacity to detoxify RO/NS [86]. These facts may explain overall better cancer survival. In CSCs, the level of RO/NS is not that high, comparatively to surrounding CCs [87–90]. There can be several reasons to that. The mitochondrial mass can be higher in CSCs or mitochondrial functions (ATP production, Δ*ψm*) can be impaired. However, in the recent experiments with lung CSCs no difference in mitochondrial mass between CSCs and non-CSCs was found [91]. The Δ*ψm* level and the intracellular concentrations of ATP and ROS were also lower than in non-CSCs. Another possible scenario of low ROS in CSCs could be metabolic reprogramming, which is critical to sustain self-renewal and enhance the antioxidant defense mechanism. This fact is closely related to the adaptation of CSCs to hypoxia requiring a biochemical trim characterized by a glycolytic-oriented metabolism that counterbalances a poor mitochondrial apparatus. In metabolic shift, CSCs showed a greater reliance on glycolysis for energy supply compared with the parental cells [92]. On the other hand, ALDH are a group of enzymes that oxidize aldehydes formed in the process of alcohol metabolism. High levels of the detoxifying enzyme ALDH1 were frequently associated with CSCs, and this marker was used for the identification of CSCs [93]. Recently, Honoki et al. evaluated the cancer spheroid subpopulation of cells from human sarcoma with high ALDH1 activity and found that these cells possess strong chemoresistance and detoxifying capability [94]. The identification of CSCs from human lung CCs identified cells with high ALDH1 activity, which was attributed to high self-renewal capacity, differentiation, and resistance to chemotherapy [95]. Breast CSCs identified as ALDH1-positive play a significant role in resistance to chemotherapy [96]. It seems like ALDH protects the drug-tolerant subpopulation of cells, including CSCs, from the potentially toxic effects of elevated levels of RO/NS. Not surprisingly, pharmacologic disruption of ALDH activity leads to accumulation of ROS to toxic levels, even within the drug-tolerant subpopulation [97].

## 6. Suggested Principles of Drugs Design towards CSCs Resistance

Some physiological metabolites such as pyruvate, tetrahydrofolate, and glutamine act as powerful cytotoxic agents on CSCs when supplied at doses that perturb the biochemical network, sustaining the resumption of aerobic growth after the hypoxic dormant state [98]. This indicates that the metabolic state of CSCs must be crucial for their resistant to therapy because when CSCs need to differentiate and proliferate, they shift from anaerobic to aerobic status.

The principles of drug resistance in CCs can be also applicable to CSCs. Cells can be resistant to the drug by (1) active drug efflux by drug transporters, such as Pgp, MRP, and BCRP; (2) loss of cell surface receptors and/or drug transporters or alterations in membrane lipid composition; (3) compartmentalization of the drug in cellular vesicles; (4) altered/increased drug targets; (5) metabolic disruption due to OXPHOS; (6) alterations in cell cycle; (7) increased drug metabolism/enzymatic inactivation; (8) active damage repair; (9) inhibition of apoptotic pathways. However, targeting RO/NS upon designing novel therapeutic strategy to overcome chemo- and/or radioresistance of CCs is associated with some difficulties and should be considered with extra care. This is because antioxidant systems not only remove oxidants but also maintain them at an optimum level [99]. Therefore, besides obvious pharmacological properties (low toxicity, subnanomolar active concentrations, solubility, and oral bioavailability), the following principles should be taken into account when rationally designing such drugs: (i) they should transiently interact with proteins that block autophagy or promote apoptosis to allow sufficient RO/NS accumulation; (ii) ideally, those drugs should have an antagonist with higher affinity to the drug and lower affinity to surrounding molecules; (iii) specific moiety for selective delivery to these organelles should be considered; (iv) low adverse side effects should be taken into account. Although a number of drugs triggering apoptotic or autophagic events have been produced for the treatment of cancer, only few of them can meet the above criteria and are summarized in Table 1. In addition, few other drugs have to be added. Alpha-tocopheryl succinate (*α*-TOS), an anionic analogue of vitamin E [100] of which mechanism of action involves interaction with ubiquinone-binding site of mitochondrial complex II and concomitant inhibition of succinate dehydrogenase (SDH) activity [101]. It is accompanied by recombination with molecular oxygen to yield ROS and permeabilization of mitochondria [102]. Proapoptotic drug BMD188 (cis-1-hydroxy-4-(1-naphthyl)-6-octylpiperidine-2-one) generates mitochondrial ROS and triggers apoptosis by activation of caspase-3. It was reported to inhibit the primary growth of prostate CSCs [41, 103]. Antineoplastic drug LND, ionidamine, 1-(2,4-dichlorobenzyl)-1H-indazole-3-carboxylic acid has been shown to inhibit glycolysis and induce mitochondria-mediated apoptosis by activation of caspase-9, caspase-3, and Akt/mTOR pathways [104]. Natural terpenoid aldehyde gossypol has been shown to increase ROS and induce apoptosis and necrosis via inhibition of Bcl-2, activation of caspase-3, cytochrome c release from mitochondria, and displacing BH3-only proteins from Bcl-2 [42]. Both gossipol and its derivative, apogossypolone (ApoG2), were also shown to induce autophagy in several CCs through Beclin-1-mediated ROS upregulation [105–108]. Polyunsaturated fatty acids (PUFAs) induce apoptosis and autophagy by means of mitochondrial ROS-mediated Akt-mTOR signaling [43, 109]. Finally, inhibitors of oxidoreductase thioredoxin (TrxR) scavenging ROS provide a promising therapeutic target for CSCs intervention. In particular, Ru(II) polypridyl complexes inducing ROS-mediated apoptosis have been suggested [44]. Organoselenium compound BBSKE (1,2-[bis(1,2-benzisoselenazolone-3(2H)-ketone)]ethane) is a TrxR inhibitor which induce apoptosis via Bcl-2/Bax pathway. Since TrxR mediates resistance to irradiation of a non-small cell lung cancer, BBSKE has been proposed as a radiosensitizer in some clinical trials [110].

## 7. Concluding Remarks

It is becoming clearer that a single drug against cancer would not be effective to cure the disease as CCs learn how to become resistant in the middle-long term along the treatment and persist hidden in the body of cancer patients upon reactivation. In principle, conventional treatments are effective to induce apoptosis or autophagy in the bulk of the tumor particularly on CCs but without affecting the CSCs. The fatal consequences of this are not only that conventional therapy favors the presence of the CSC but also that they resume growth more aggressively. The reasons of CSCs resistance to the induction of apoptosis, autophagy, or hypoxia are closely related to their metabolic status which in turn depends on mitochondria as the main source of energy. A synthetic lethality or combinatorial therapy followed by animal studies to specify the dose and timing and minimize side effects should be considered for effective targeting of CSCs.

## Figures and Tables

**Table 1:** Drugs targeting specific CSCs and their modes of action.

Compounds	Mechanism of action	Type of CSCs	References
Temozolomide (TMZ)	Reversal of EMT and chemosensitizing CSCs	Glioblastoma	[27]
STA-4783	Targets OS by Hsp70 induction and induces ROS within CCs	Breast	[28]
Geldanamycin	Inhibition of the TRAP1 chaperone function	Breast, lung, and neural	[31]
Rottlerin (ROT)	Inhibitor of PI3K/Akt/mTOR pathway and inducer of apoptosis	Pancreatic	[33]
Bardoxolone methyl	Nrf2 inhibitor	Leukemia	[34]
Resveratrol	Wnt inhibitor	Breast	[35]
Honokiol	Notch inhibitor	Melanoma	[36]
Chloroquine (CQ)	Autophagy inducer	Colorectal	[37]
Pemetrexed	Folate antagonist	Lung	[38]
Melatonin	Induces autophagy by increasing ROS	Glioma	[39]
Salinomycin	K+-ionophore and triggers autophagy	General CSCs	[40]
BMD188	Proapoptotic	Prostate cancer	[41]
Gossypol	Hsp70 induction, ROS induction	Breast, leukemia	[42]
PUFAs	Induces apoptosis and autophagy	Colorectal	[43]
TrxR	ROS scavenger	Cervical	[44]

**Table 2:** Difference in CSCs and CS survival after chemo-/radiotherapy. Higher apoptosis, autophagy/mitophagy, ROS, and lower metabolic activity in CSCs versus CSs may be predominant factors in explaining chemo-/radiotherapy.

Survival properties	CCs	CSCs
RO/NS	↑	↓
Metabolic activity	↑	↓
Autophagy/mitophagy	↓	↑
Apoptosis	↓	↑
Chemo-/radioresistance	↑	↓

## Abbreviations

Δ*ψm*: Mitochondrial membrane potential
CCs: Cancer cells
CSCs: Cancer stem cells
EMT: Epithelial-Mesenchymal Transition
GSH: Glutathione
GSSG: Oxidised glutathione
mTOR: Mammalian target of rapamycin
OS: Oxidative stress
RO/NS: Reactive oxygen/nitrogen species
ROS: Reactive oxygen species.
